# Supplementary material for: Multivalent bicyclic peptides are an effective antiviral modality that can potently inhibit SARS-CoV-2
Source: Nat Commun. 2023 Jun 16;14:3583. doi: 10.1038/s41467-023-39158-1 (PMC10275983; doi:10.1038/s41467-023-39158-1)

Supplementary Information

Multivalent bicyclic peptides are an effective antiviral modality that can potently inhibit SARS-CoV-2

Katherine U Gaynor, Marina Vaysburd, Maximilian A J Harman, Anna Albecka, Phillip Jeffrey, Paul Beswick, Guido Papa, Liuhong Chen, Donna Mallery, Brian McGuinness, Katerine Van Rietschoten, Steven Stanway, Paul Brear, Aleksei Lulla, Katarzyna Ciazynska, Veronica T Chang, Jo Sharp, Megan Neary, Helen Box, Jo Herriott, Edyta Kijak, Lee Tatham, Eleanor G Bentley, Parul Sharma, Adam Kirby, Ximeng Han, James P Stewart, Andrew Owen, John A. G. Briggs, Marko Hyvönen, Michael J Skynner & Leo C James

Contents

3-5 Supplementary Table 1: Aligned sequences from phage sanger sequencing.

6 Supplementary Table 2: Domain mapping of *Bicycles* against Spike by SPR.

7 Supplementary Table 3: AlphaScreen competition binding to determine which *Bicycles* target overlapping epitopes.

8 Supplementary Table 4: AlphaScreen competition assay to determine ability of *Bicycles* to inhibit ACE2 binding.

9 Supplementary Table 5: Data collection and refinement statistics.

10 Supplementary Table 6: AlphaScreen competition assay to determine ability of multimerized E2 *Bicycles* to inhibit ACE2 binding.

11-12 Supplementary Table 7: List of *Bicycle* IC90s.

13 Supplementary Figure 1: Immunofluorescence data of *Bicycles* inhibition of SARS-CoV-2 infection and cell-cell fusion.

14 Supplementary Figure 2: Summary of intravenous (i.v.) and sub-cutaneous (s.c.) pharmacokinetics for E2 trimer and E2E4 biparatopic.

15 Supplementary Figure 3: Pharmacokinetic-pharmacodynamic (PKPD) models.

Supplementary Table 1: Aligned sequences from phage sanger sequencing.
Peptide sequences from selection against Spike, organized by epitope group.

Epitope	Scaffold	Sequence
1*	TATA	A C **E** **Y** V **G** **P** M C Y **R** **L** **Y** C A
1	TATA	A C **E** **Y** N **G** **P** Y C Y **R** **L** **Y** C A
1	TATA	A C **E** **Y** Q **G** **P** H C Y **R** **L** **Y** C A
2*	TCMT	A C **P** Y V A **G** R **G** T C **L** **L** C A
2	TCMT	A C **P** F K P **G** V **G** T C **L** **L** C A
2	TCMT	A C **P** F P P **G** M **G** T C **L** **L** C A
2	TCMT	A C **P** H M P **G** S **G** T C **L** **L** C A
2	TCMT	A C **P** H P P **G** R **G** T C **L** **L** C A
2	TCMT	A C **P** H Q P **G** F **G** T C **L** **L** C A
2	TCMT	A C **P** W E A **G** K **G** T C **L** **L** C A
2	TCMT	A C **P** Y A P **G** M **G** T C **L** **L** C A
2	TCMT	A C **P** Y A P **G** N **G** T C **L** **L** C A
2	TCMT	A C **P** Y L A **G** T **G** T C **L** **L** C A
2	TCMT	A C **P** Y N A **G** T **G** T C **L** **L** C A
2	TCMT	A C **P** Y N K **G** E **G** T C **L** **L** C A
2	TCMT	A C **P** Y Q P **G** S **G** T C **L** **L** C A
2	TCMT	A C **P** Y R E **G** T **G** T C **L** **L** C A
2	TCMT	A C **P** Y S P **G** Q **G** T C **L** **L** C A
2	TCMT	A C **P** Y S P **G** S G T C **L** **L** C A
2	TCMT	A C L Y P P **G** K G T C **L** **L** C A
2	TCMT	A C **P** S P A **G** R G T C **L** **L** C A
2b*	TATA	A C **M** F V **P** C A V **R** H A **L** **G** **L** C A
2b	TATA	A C **M** F T **P** C H V **R** E I **L** **G** **L** C A
2b	TATA	A C **M** F V **P** C A A **R** H E **L** **G** **L** C A
2b	TATA	A C **M** F V **P** C A A **R** V E **L** **G** **L** C A
2b	TATA	A C **M** F V **P** C A I **R** Q T **L** **G** **L** C A
2b	TATA	A C **M** F V **P** C A T **R** H E **L** **G** **L** C A
2b	TATA	A C **M** F V **P** C A T **R** H Q **L** **G** **L** C A
2b	TATA	A C **M** F V **P** C A T **R** H S **L** **G** **L** C A
2b	TATA	A C **M** F V **P** C A T **R** L A **L** **G** **L** C A
2b	TATA	A C **M** F V **P** C A T **R** L Q **L** **G** **L** C A
2b	TATA	A C **M** F V **P** C A T **R** Q E **L** **G** **L** C A
2b	TATA	A C **M** F V **P** C A T **R** Q M **L** **G** **L** C A
2b	TATA	A C **M** F V **P** C A T **R** V A **L** **G** **L** C A
2b	TATA	A C **M** F V **P** C A V **R** E E **L** **G** **L** C A
2b	TATA	A C **M** F V **P** C A V **R** E I **L** **G** **L** C A
2b	TATA	A C **M** F V **P** C A V **R** H S **L** **G** **L** C A
2b	TATA	A C **M** F V **P** C A V **R** K D **L** **G** **L** C A
2b	TATA	A C **M** F V **P** C A V **R** Q T **L** **G** **L** C A
2b	TATA	A C **M** G V **P** C K V **R** E I **L** **G** **L** C A
3*	TATA	A C E D N D **W** V **Y** C **S** **T** C A
3	TATA	A C E D H D **W** V **Y** C **S** **T** C A
3	TATA	A C E S N D **W** V **Y** C **S** **T** C A
3	TATA	A C L D E T **W** I **Y** C **S** **T** C A
3	TATA	A C P D E T **W** V **Y** C **S** **T** C A

3	TATA	A C P D V S **W** I **Y** C **S** **T** C A
3	TATA	A C E Q N G **W** I **Y** C **S** **T** C A
3	TATA	A C P N I S **W** I **Y** C **S** **T** C A
3	TATA	A C T D R S **W** I F C **S** **T** C A
3	TATA	A C A P T S G **W** I **Y** C **S** **T** C A
3	TATA	A C G R D S S **W** I **Y** C **S** **T** C A
3	TATA	A C P E A N S **W** V **Y** C **S** **T** C A
3	TATA	A C R G T P A **W** K A C A I C A
4*	TATB	A C I P L **D** **W** **T** C M I A C A
4	TATA	A C K I H **D** **W** **T** C L L R C A
4	TATB	A C **D** **W** **T** C Y F R P L P C A
4	TATB	A C **D** **W** **T** C Y L R P L P C A
4	TATB	A C **D** **W** **T** C Y L T M M P C A
4	TATB	A C **D** **W** **T** C Y M S M K P C A
4	TATB	A C **D** **W** **T** C Y I S P M F D C A
4	TATB	A C **D** **W** **T** C Y L N I Y H E C A
4	TATB	A C **D** **W** **T** C Y L R I H E A C A
4	TATB	A C **D** **W** **T** C Y M D Y L S N C A
4	TATB	A C **D** **W** **T** C Y M R I N D A C A
4	TCMT	A C **D** **W** **T** C Y I N I Y N T C A
4	TCMT	A C F D **D** **W** **T** C Y I Q M C A
5*	TATB	A C A N P D **N** **P** **V** C **R** **F** **Y** C A
5	TATB	A C A N H D **N** **P** **V** C **R** **F** **Y** C A
5	TATB	A C A S P D **N** **P** **V** C **R** **F** **Y** C A
5	TATB	A C E N M D **N** **P** **V** C **R** **F** **Y** C A
5	TATB	A C F N I D **N** **P** **V** C **R** **F** **Y** C A
5	TATB	A C H N L E **N** **P** **V** C **R** **F** **Y** C A
5	TATB	A C H N P S **N** **P** **V** C **R** **F** **Y** C A
5	TATB	A C K N Y E **N** **P** **V** C **R** **F** **Y** C A
5	TATB	A C L N A E **N** **P** **V** C **R** **F** **Y** C A
5	TATB	A C L N K H **N** **P** **V** C **R** **F** **Y** C A
5	TATB	A C L N P E **N** **P** **V** C **R** **F** **Y** C A
5	TATB	A C L N V E **N** **P** **V** C **R** **F** **Y** C A
5	TATB	A C M N A A **N** **P** **V** C **R** **F** **Y** C A
5	TATB	A C M N E D **N** **P** **V** C **R** **F** **Y** C A
5	TATB	A C M N P D **N** **P** **V** C **R** **F** **Y** C A
5	TATB	A C M N T D **N** **P** **V** C **R** **F** **Y** C A
5	TATB	A C N N P A **N** **P** **V** C **R** **F** **Y** C A
5	TATB	A C Q N P G **N** **P** **V** C **R** **F** **Y** C A
5	TATB	A C R N P E **N** **P** **V** C **R** **F** **Y** C A
5	TATB	A C S N P E **N** **P** **V** C **R** **F** **Y** C A
5	TATB	A C Y N Q E **N** **P** **V** C **R** **F** **Y** C A
6*	TATB	A C D H Y H C P W L A L G G S C A
7*	TATB	A C I N P Y C E H H I Y L E H C A
9*	TATB	A C **M** **N** **P** **F** F Y D C E T V C A
9	TATB	A C **M** **N** **P** **F** F Y D C D H I C A
9	TATB	A C **M** **N** **P** **F** F Y D C E D R C A

9	TATB	A	C	**M**	**N**	**P**	**F**	F	Y	D	C	E	E	I	C	A		
9	TATB	A	C	**M**	**N**	**P**	**F**	F	Y	D	C	E	N	P	C	A		
9	TATB	A	C	**M**	**N**	**P**	**F**	F	Y	D	C	E	R	T	C	A		
9	TATB	A	C	**M**	**N**	**P**	**F**	F	Y	D	C	E	Y	V	C	A		
9	TATB	A	C	**M**	**N**	**P**	**F**	F	Y	D	C	H	E	Q	C	A		
9	TATB	A	C	**M**	**N**	**P**	**F**	F	Y	D	C	K	V	V	C	A		
9	TATB	A	C	**M**	**N**	**P**	**F**	Y	Y	D	C	E	E	V	C	A		
11*	TCMT	A	C	**F**	**P**	E	**P**	**W**	**L**	**G**	**L**	C	**T**	**P**	C	A		
11	TCMT	A	C	**F**	**P**	A	**P**	**W**	**L**	**G**	**L**	C	**T**	**P**	C	A		
12*	TATA	A	C	**S**	S	K	**F**	C	D	**A**	**W**	W	N	**F**	**N**	R	C	A
12	TATA	A	C	**S**	D	A	**F**	C	S	**A**	**W**	W	G	**F**	**N**	Q	C	A
12	TATA	A	C	**S**	D	D	**F**	C	S	**A**	**W**	W	G	**F**	**N**	H	C	A
12	TATA	A	C	**S**	D	E	**F**	C	S	**A**	**W**	W	G	**F**	**N**	E	C	A
12	TATA	A	C	**S**	N	K	**F**	C	D	**A**	**W**	W	N	**F**	**N**	R	C	A

* denotes the lead sequence as described in Table 1. Note for Epitope 6 and 7, these sequences appeared as "singletons" with no similar motifs appearing in outputs.

text in bold denotes conserved residues

Supplementary Table 2: Domain mapping of *Bicycles* against Spike by SPR. *Bicycle* representatives of each epitope were screened against six protein constructs; Spike Trimer, S1, S1-RBD, S1-NTD, S2 and ACE2. The equilibrium dissociation constant (K_D) is displayed as a geometric mean (geomean) value (N=3) with upper (UCI) and lower (LCI) confidence intervals.

Bicycle (Epitope)	Protein Construct	Geomean K_D (nM)	Lower 95% CI (nM)	Upper 95% CI (nM)	N
BCY17548 (E1)	Spike Trimer	6200	8200	4600	3
	S1	5100	15000	1800	3
	S1-RBD	3600	5000	2600	3
	S1-NTD	>	-	-	3
	S2	>	-	-	3
	ACE2	>	-	-	3
BCY16591 (E2)	Spike Trimer	730	1100	490	3
	S1	830	1100	620	3
	S1-RBD	650	1300	310	3
	S1-NTD	>	-	-	3
	S2	>	-	-	3
	ACE2	>	-	-	3
BCY17543 (E3)	Spike Trimer	5100	15000	1700	3
	S1	2500	7000	910	3
	S1-RBD	2300	4500	1200	3
	S1-NTD	>	-	-	3
	S2	>	-	-	3
	ACE2	>	-	-	3
BCY18150 (E9)	Spike Trimer	3100	3300	2800	3
	S1	3800	6000	2400	3
	S1-RBD	4100	7300	2300	3
	S1-NTD	>	-	-	3
	S2	>	-	-	3
	ACE2	>	-	-	3
BCY15466 (E4)	Spike Trimer	110	290	42	3
	S1	2900	28000	300	3
	S1-RBD	>	-	-	3
	S1-NTD	440	1000	190	3
	S2	>	-	-	3
	ACE2	>	-	-	3
BCY16107 (E6)	Spike Trimer	970	3800	250	3
	S1	>	-	-	3
	S1-RBD	>	-	-	3
	S1-NTD	3900	6100	2500	3
	S2	>	-	-	3
	ACE2	>	-	-	3
BCY16115 (E7)	Spike Trimer	3200	3800	2700	3
	S1	>	-	-	3
	S1-RBD	>	-	-	3
	S1-NTD	4000	6400	2500	3
	S2	>	-	-	3
	ACE2	>	-	-	3
BCY16903 (E5)	Spike Trimer	15	150	1.6	2
	S1	24	240	2.4	2
	S1-RBD	>	-	-	3
	S1-NTD	>	-	-	3
	S2	>	-	-	3
	ACE2	>	-	-	3
BCY19905 (E11)	Spike Trimer	450	1000	190	3
	S1	>	-	-	3
	S1-RBD	>	-	-	3
	S1-NTD	>	-	-	3
	S2	530	780	360	3
	ACE2	>	-	-	3
BCY19901 (E12)	Spike Trimer	610	700	530	3
	S1	>	-	-	3
	S1-RBD	>	-	-	3
	S1-NTD	>	-	-	3
	S2	810	1200	530	3
	ACE2	>	-	-	3

Supplementary Table 3: AlphaScreen competition binding to determine which *Bicycles* target overlapping epitopes. Unmodified *Bicycle* representatives of each epitope were competed against representative biotinylated *Bicycles* of each epitope binding a certain Spike protein construct dependent on the domain mapping profile. The half maximal inhibitory concentration (IC_{50}) is displayed as the arithmetic mean average of two technical replicates.

Bicycle ID (Epitope)	Biotinylated Bicycle (Epitope)	Spike Domain	IC_{50} (μM)	n
BCY15354 (E1)	BCY15762 (E1)	S1	0.36	2
BCY16591 (E2)			0.11	2
BCY15231 (E3)			0.31	2
BCY16207 (E9)			-	2
BCY15354 (E1)	BCY15760 (E2)	S1	1.6	2
BCY16591 (E2)			0.35	2
BCY15231 (E3)			-	2
BCY16207 (E9)			-	2
BCY15354 (E1)	BCY15763 (E3)	S1	0.55	2
BCY16591 (E2)			-	2
BCY15231 (E3)			0.40	2
BCY16207 (E9)			1.1	2
BCY16107 (E6)	BCY16257 (E6)	S-Trimer	0.20	2
BCY16115 (E7)			-	2
BCY16107 (E6)	BCY16274 (E7)	S-Trimer	-	2
BCY16115 (E7)			1.0	2
BCY19905 (E11)	BCY19320 (E11)	S2	0.064	2
BCY19901 (E12)			-	2
BCY19905 (E11)	BCY19908 (E12)	S2	-	2
BCY19901 (E12)			0.22	2

Supplementary Table 4: AlphaScreen competition assay to determine ability of *Bicycles* to inhibit ACE2 binding. Unmodified monomeric *Bicycle* representatives of each epitope were competed against Spike Trimer:ACE2 binding interaction. The half maximal inhibitory concentration (IC_{50}) is displayed as a geometric mean (geomean) value (minimum N=2) with upper (UCI) and lower (LCI) confidence intervals. A non-Spike binding Bicycle was used as a negative control.

Bicycle ID	Epitope	Multimeric State	Geomean IC_{50} (nM)	Lower 95% CI (nM)	Upper 95% CI (nM)	N
-ve Control	Non-binder	Monomer	>21000	-	-	6
BCY17548	E1	Monomer	>20000	-	-	2
BCY16591	E2	Monomer	520	360	750	4
BCY17543	E3	Monomer	>20000	-	-	2
BCY15446	E4	Monomer	>16000	-	-	2
BCY16903	E5	Monomer	>20000	-	-	2
BCY16107	E6	Monomer	>20000	-	-	2
BCY16115	E7	Monomer	>14000	-	-	2
BCY18150	E9	Monomer	>20000	-	-	2
BCY19905	E11	Monomer	>20000	-	-	2
BCY19901	E12	Monomer	>20000	-	-	2

Supplementary Table 5: Data collection and refinement statistics. RBD complexes with *Bicycle* ligands. Statistics for both data integration and model refinement are given (values for the highest resolution shell are given in parentheses).

	sRBD:E2	sRBD:E2b
PDB code	7Z8O	8AAA
**Data collection**		
Wavelength (Å)	0.8	0.98
Temperature (K)	100	100
Beamline	Diamond Light Source I04	Diamond Light Source I04
Detector	Eiger2 XE 16M	Eiger2 XE 16M
Rotation per image (°)	0.1	0.05
Total rotation range (°)	300	360
Exposure time per image (s)	0.05	0.02
Space group	$P2_12_12_1$	P32 21
a, b, c (A)	44.2 55.7 82.7	112.2 112.2 35.5
α, β, γ (°)	90, 90, 90	90, 90, 120
Resolution range	46.2 - 0.96 (0.98 - 0.96)	56.1 - 1.9 (1.97 - 1.90)
Total No of reflections	2313853 (34008)	266723 (10644)
No of unique reflections	123986 (5517)	13749 (686)
Completeness	99.2 (90.4)	91.5 (65.2)
Multiplicity	18.7 (6.2)	19.4 (15.5)
<I>/<σ(I)>	19.6 (0.9)	9.3 (1.9)
R_{merge}	0.06 (1.51)	0.25 (1.68)
$CC_{1/2}$	1.00 (0.37)	0.99 (0.86)
**Refinement**		
Rfactor	0.1191 (0.342)	0.181 (0.394)
R-free	0.1334 (0.301)	0.2276 (0.3432)
R.m.s. deviations (angle, °)	1.99	0.94
R.m.s. deviations (length, Å)	0.017	0.008
Ramachandran plot		
Ramachandran favored (%)	97.47	95.63
Ramachandran allowed (%)	2.53	4.37
Ramachandran outliers (%)	0	0
Average B-factor	16.4	35.8
macromolecules	14.2	35.8
ligands	15.3	35.1
solvent	28	35.5

Supplementary Table 6: AlphaScreen competition assay to determine ability of multimerized E2 *Bicycles* to inhibit ACE2 binding. E2 *Bicycle* of constant sequence and variable multimeric state were competed against Spike Trimer:ACE2 binding interaction. The half maximal inhibitory concentration (IC_{50}) is displayed as a geometric mean (geomean) value (minimum N=2) with upper (UCI) and lower (LCI) confidence intervals. A non-Spike binding Bicycle was used as a negative control. The lower limit of quantification (LoQ) was defined as [Spike Protein]/2, in this case equal to 0.1 nM.

Bicycle ID	Epitope	Multimeric State	Geomean IC_{50} (nM)	Lower 95% CI (nM)	Upper 95% CI (nM)	N
-ve Control	Non-binder	Monomer	>21000	-	-	6
BCY16591	E2	Monomer	520	360	750	4
BCY17023	E2	Homodimer	0.74	0.047	12	2
BCY17021	E2	Homotrimer	0.075*	0.014	0.42	2
BCY17022	E2	Homotetramer	0.096*	0.094	0.097	2

*LoQ = 0.1 nM

Supplementary Table 7: List of *Bicycle* IC90s. IC90 values are given by figure panel for each Bicycle as calculated from each dose-response fit.

Figure 2B	
**Bicycle**	**IC90 (M)**
E2 Monomer	1.6×10^{-2}
E2 Dimer	7.3×10^{-5}
E2 Trimer	7.2×10^{-7}
E2 Tetramer	4.3×10^{-7}
**Figure 2C**	
**Bicycle**	**IC90 (M)**
PEG1	2.6×10^{-8}
PEG5	5.3×10^{-8}
PEG10	4.9×10^{-8}
PEG23	1.3×10^{-7}
**Figure 2D**	
**Bicycle**	**IC90 (M)**
E3 Trimer	8.3×10^{-6}
E5 Trimer	2.2×10^{-6}
**Figure 2E**	
**Bicycle**	**IC90 (M)**
E1E2	7.6×10^{-4}
E2E3	1.6×10^{-6}
E1E5	8.3×10^{-6}
E2E5	1.8×10^{-7}
E3E5	1.1×10^{-6}
**Figure 2G**	
**Bicycle**	**IC90 (M)**
E1E4	6.3×10^{-6}
E2E4	8.3×10^{-8}
E3E4	1.9×10^{-6}
E4E5	2.4×10^{-6}
**Figure 3A**	
**Bicycle**	**IC90 (M)**
E2 Trimer	3.9×10^{-9}
E2E4	8.1×10^{-9}
**Figure 3B**	
**Bicycle**	**IC90 (M)**
E2	2.1×10^{-7}
E2E4	8.9×10^{-7}
**Figure 3C**	
**Bicycle**	**IC90 (M)**
E2	2.56×10^{-8}
E2E4	2.17×10^{-8}
**Figure 4B**	

Bicycle	IC90 (M)
Beta	2.7×10^{-4}
Beta 484	7.3×10^{-8}
Delta	1.4×10^{-4}
Delta 452	5.1×10^{-9}
**Figure 4C**	
**Bicycle**	**IC90 (M)**
E3E5	1.7×10^{-7}
E4E5	5.5×10^{-8}
E2c Trimer	2.0×10^{-7}
E2E4	1.3×10^{-7}
**Figure 4D**	
**Bicycle**	**IC90 (M)**
E3E5	1.2×10^{-6}
E4E5	6.0×10^{-7}
E2c Trimer	1.3×10^{-6}
E2E4	1.8×10^{-6}
**Figure 4E**	
**Bicycle**	**IC90 (M)**
E3E5	4.9×10^{-6}
E4E5	2.0×10^{-4}
E2c Trimer	5.9×10^{-6}
E2E4	9.7×10^{-5}
**Figure 4F**	
**Bicycle**	**IC90 (M)**
E2E4	2.1×10^{-7}
**Figure 4G**	
**Bicycle**	**IC90 (M)**
E3E5	3.3×10^{-5}
E2c Trimer	1.6×10^{-5}

Supplementary Figure 1: Immunofluorescence data of *Bicycles* inhibition of SARS-CoV-2 infection and cell-cell fusion. (a) Quantification of NP expression in Vero TMPRSS2/ACE2 cells 18 hours post-infection with SARS-CoV-2 (Wuhan-Hu-1 strain) in the presence of homotrimeric E2 or biparatopic E2E4. Cells were fixed and stained with anti-NP antibody (CAT). A representative image of at least 3 biological replicates is shown. Data are presented as mean values +/- SEM. Ordinary one-way ANOVA was used for statistical analysis and significance indicated with respect to DMSO condition. **(b)** Immunofluorescence images of GFP positive syncytia 12 hours post-Spike transfection in the presence of E2E4 and E2 trimer *Bicycles* at a range of concentrations. Quantification of this data is shown in Figure 3E. Scale bar is 200 µM.

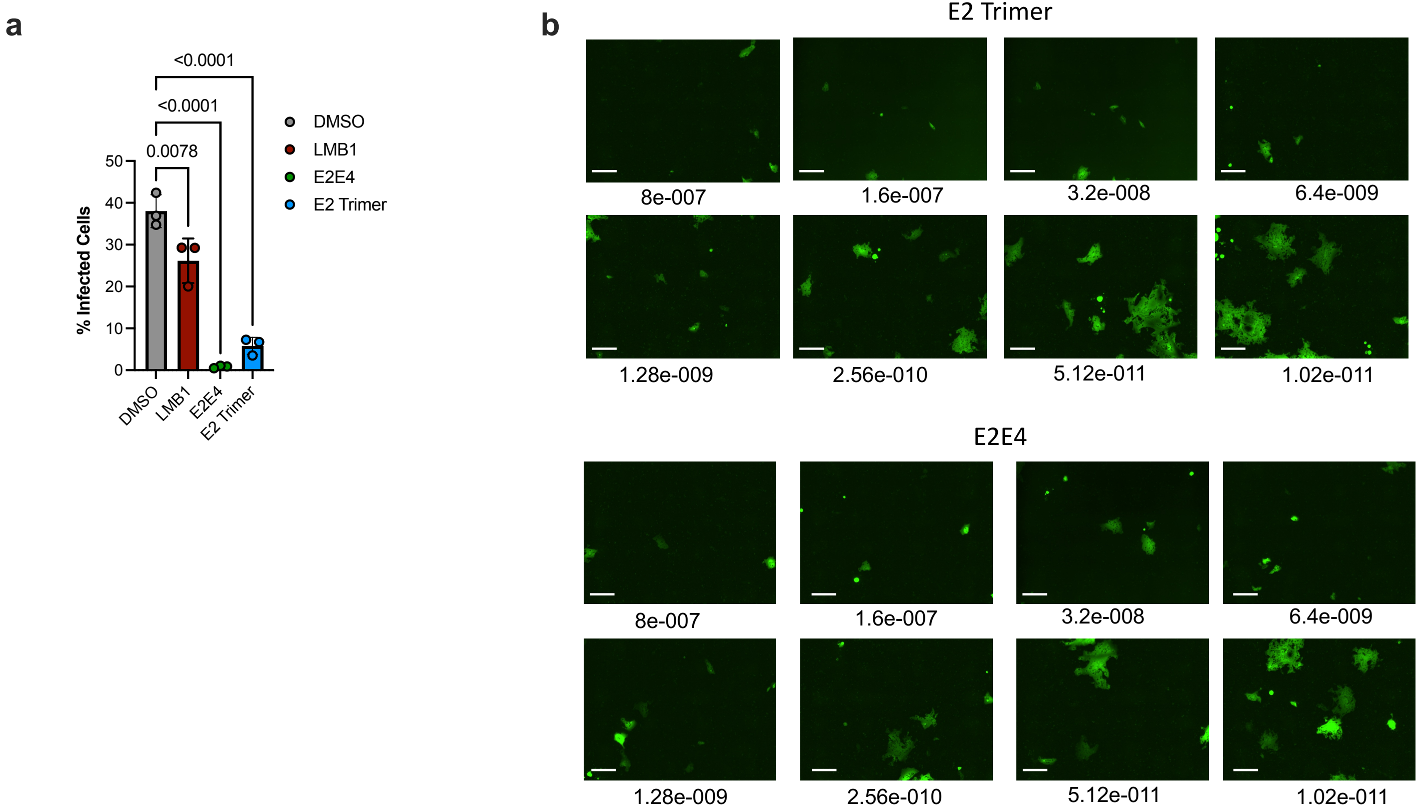

Supplementary Figure 2: Summary of intravenous (i.v.) and sub-cutaneous (s.c.) pharmacokinetics for E2 trimer and E2E4 biparatopic. (A,B,C,D) *Bicycle* multimers were administered to both mice and hamsters (N=6, alternate composite sampling resulting in N=3 per timepoint) via i.v. and s.c. routes of administration. *Bicycle* plasma concentration over time data displayed as mean +/- standard deviation (SD) for N=3 biological replicates. **(E)** Pharmacokinetic parameters were subsequently evaluated.

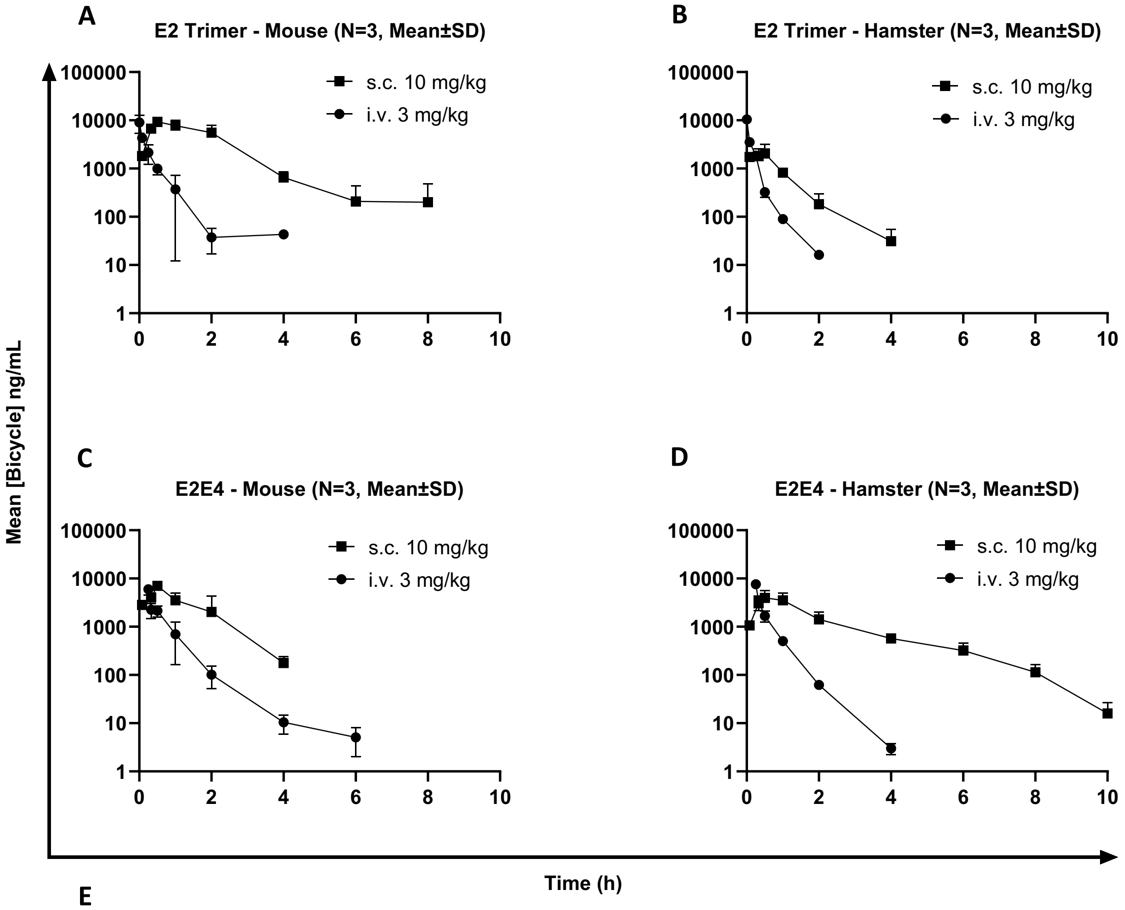

E

Compound	BCY17021				BCY18656			
Species	Mouse		Hamster		Mouse		Hamster	
Route of Administration	i.v.	s.c.	i.v.	s.c.	i.v.	s.c.	i.v.	s.c.
Nominal Dose (mg/kg)	3.0	10	3.0	10	3.0	10	3.0	10
C_{max} (ng/mL)	9000	9300	10000	2100	6000	7000	7600	4300
T_{max} (h)	0.0	0.5	0.0	0.5	0.25	0.5	0.25	0.6
$T_{1/2}$ (h)	0.6	1.2	0.4	0.7	0.6	0.7	0.4	1.1
Vd_{ss} (L/kg)	0.45	-	0.27	-	0.64	-	0.46	-
Cl (mL/min/kg)	16	-	19	-	20	-	20	-
$AUC_{0\text{-}inf}$ (ng.h/mL)	3100	19000	2600	2100	2500	8900	2500	8600
F (%)	-	NR*	-	16	-	100	-	100

*inconsistent plasma concentration-time profile, therefore bioavailability not reported (NR).

Supplementary Figure 3: Pharmacokinetic-pharmacodynamic (PKPD) models. PKPD models were used to select an appropriate in vivo dosing regimen that would sustain a minimum of 90% target coverage at trough for both the E2 Trimer and E2E4 biparatopic. Using the appropriate data the target coverage predictions are; **(A)** E2 Trimer mouse s.c. administration **(B)** E2 Trimer hamster s.c. administration, **(C)** E2E4 biparatopic mouse s.c. administration **(D)** E2E4 biparatopic hamster s.c. administration. Each panel is expressed as % Bicycle Target Coverage over time where black dotted lines reference 90% coverage at 8 hours, indicating 90% target coverage at trough based on a t.i.d. dosing regimen.

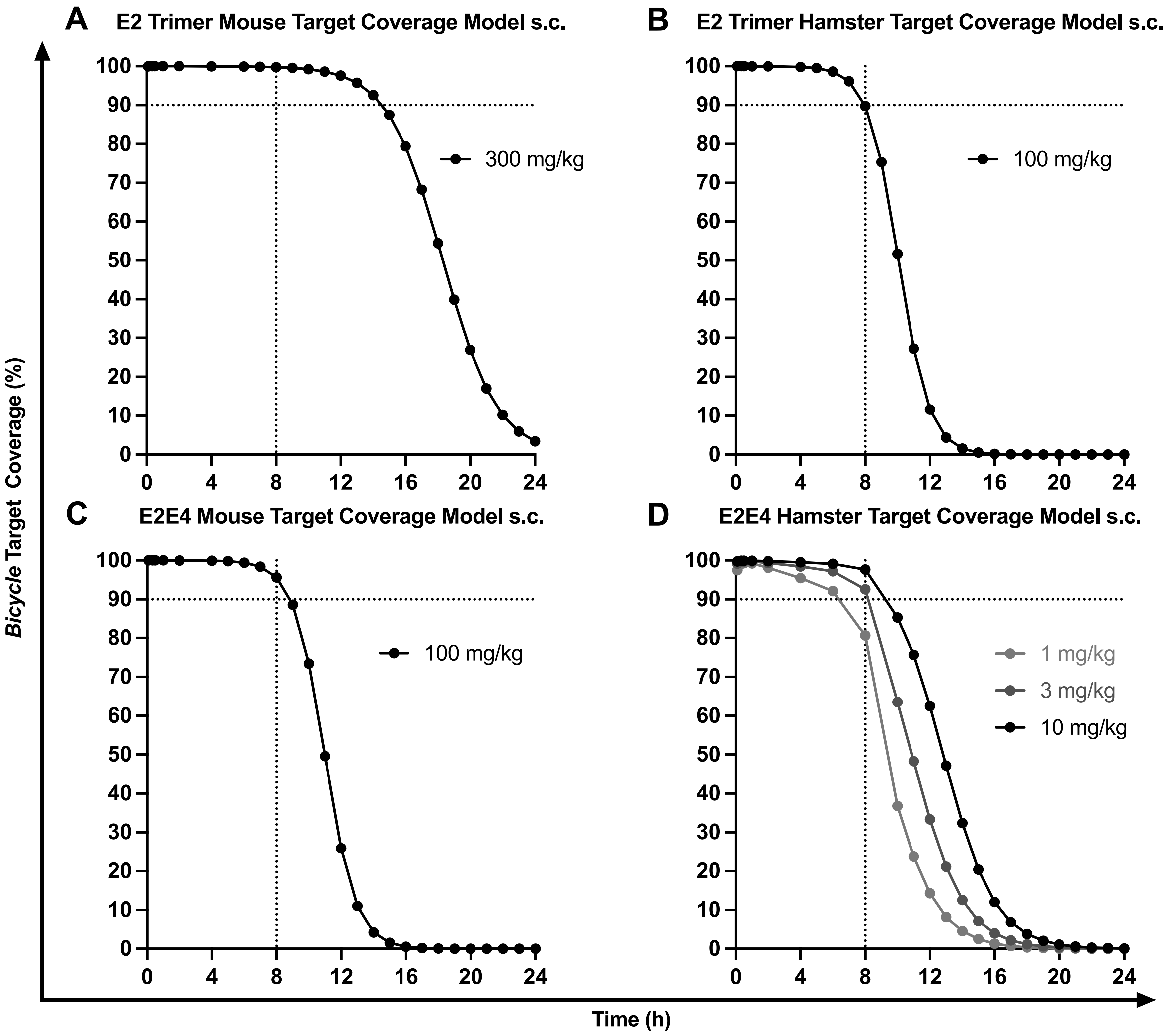

Supplement: Supplementary file 1 — Supplementary Information [file 41467_2023_39158_MOESM1_ESM.pdf]
