# Supplementary material for: Multivalent bicyclic peptides are an effective antiviral modality that can potently inhibit SARS-CoV-2
Source: Nat Commun. 2023 Jun 16;14:3583. doi: 10.1038/s41467-023-39158-1 (PMC10275983; doi:10.1038/s41467-023-39158-1)
Supplement: Supplementary file 3 — Reporting Summary [file 41467_2023_39158_MOESM3_ESM.pdf]

nature portfolio

Corresponding author(s): James LC & Skynner M

Last updated by author(s): 2022/11/16

Reporting Summary

Nature Portfolio wishes to improve the reproducibility of the work that we publish. This form provides structure for consistency and transparency in reporting. For further information on Nature Portfolio policies, see our Editorial Policies and the Editorial Policy Checklist.

Statistics

For all statistical analyses, confirm that the following items are present in the figure legend, table legend, main text, or Methods section.

n/a	Confirmed	
☐	☒	The exact sample size (*n*) for each experimental group/condition, given as a discrete number and unit of measurement
☐	☒	A statement on whether measurements were taken from distinct samples or whether the same sample was measured repeatedly
☐	☒	The statistical test(s) used AND whether they are one- or two-sided<br>*Only common tests should be described solely by name; describe more complex techniques in the Methods section.*
☒	☐	A description of all covariates tested
☐	☒	A description of any assumptions or corrections, such as tests of normality and adjustment for multiple comparisons
☐	☒	A full description of the statistical parameters including central tendency (e.g. means) or other basic estimates (e.g. regression coefficient) AND variation (e.g. standard deviation) or associated estimates of uncertainty (e.g. confidence intervals)
☐	☒	For null hypothesis testing, the test statistic (e.g. *F*, *t*, *r*) with confidence intervals, effect sizes, degrees of freedom and *P* value noted<br>*Give P values as exact values whenever suitable.*
☒	☐	For Bayesian analysis, information on the choice of priors and Markov chain Monte Carlo settings
☒	☐	For hierarchical and complex designs, identification of the appropriate level for tests and full reporting of outcomes
☒	☐	Estimates of effect sizes (e.g. Cohen's *d*, Pearson's *r*), indicating how they were calculated

Our web collection on statistics for biologists contains articles on many of the points above.

Software and code

Policy information about availability of computer code

Data collection	CCP4 Package (Winn, M. D. et al. Overview of the CCP4 suite and current developments. Acta Crystallogr D Biol Crystallogr 67, 235-242, doi:10.1107/S0907444910045749 (2011). Version 8.0<br>McCoy, A. J., Grosse-Kunstleve, R. W., Storoni, L. C. & Read, R. J. Likelihood-enhanced fast translation functions. Acta Crystallogr D Biol Crystallogr 61, 458-464, doi:10.1107/S0907444905001617 (2005). Version 3.55.1<br>Murshudov, G. N., Vagin, A. A. & Dodson, E. J. Refinement of macromolecular structures by the maximum-likelihood method. Acta Crystallogr D Biol Crystallogr 53, 240-255, doi:10.1107/S0907444996012255 (1997). Version 6<br>59 Emsley, P. & Cowtan, K. Coot: model-building tools for molecular graphics. Acta Crystallogr D Biol Crystallogr 60, 2126-2132, doi:10.1107/S0907444904019158 (2004). Version 0.9.4.1<br>Long, F. et al. AceDRG: a stereochemical description generator for ligands. Acta Crystallogr D Struct Biol 73, 112-122, doi:10.1107/S2059798317000067 (2017). Version 1.0
Data analysis	Prism Version 9

For manuscripts utilizing custom algorithms or software that are central to the research but not yet described in published literature, software must be made available to editors and reviewers. We strongly encourage code deposition in a community repository (e.g. GitHub). See the Nature Portfolio guidelines for submitting code & software for further information.

Data

Policy information about availability of data

All manuscripts must include a data availability statement. This statement should provide the following information, where applicable:

- Accession codes, unique identifiers, or web links for publicly available datasets
- A description of any restrictions on data availability
- For clinical datasets or third party data, please ensure that the statement adheres to our policy

All data are available in the manuscript, in accompanying figures and tables and in the accompanying Source Data file, with the exception of two x-ray structures that have been deposited in the PDB database (https://www.rcsb.org/) with codes 7Z8O and 8AAA and also three previously deposited structures from the PDB database with codes 6M0J (https://www.rcsb.org/structure/6m0j), 7A98 (https://www.rcsb.org/structure/7A98) and 6ZP0 (https://www.rcsb.org/structure/6ZP0).

Human research participants

Policy information about studies involving human research participants and Sex and Gender in Research.

Reporting on sex and gender	Not Applicable
Population characteristics	Not Applicable
Recruitment	Not Applicable
Ethics oversight	Not Applicable

Note that full information on the approval of the study protocol must also be provided in the manuscript.

Field-specific reporting

Please select the one below that is the best fit for your research. If you are not sure, read the appropriate sections before making your selection.

☒ Life sciences ☐ Behavioural & social sciences ☐ Ecological, evolutionary & environmental sciences

For a reference copy of the document with all sections, see nature.com/documents/nr-reporting-summary-flat.pdf

Life sciences study design

All studies must disclose on these points even when the disclosure is negative.

Sample size	No statistical method was used to predetermine the sample size. Sample sizes in this study were used based on previous experience that showed significance (PMID: 34331769). For in vitro studies, the experiments were repeated at least 3 times with samples size at least 3 to control technical variations.
Data exclusions	There were no data exclusions.
Replication	Data was collected with technical replicates and experiments were repeated three times to obtain biological replicates and all replicates were successful.
Randomization	For animal studies, mice were randomly assigned to treatment groups in an age and weight-matched distribution.
Blinding	No blinding was performed because studies were independently corroborated by multiple investigators.

Reporting for specific materials, systems and methods

We require information from authors about some types of materials, experimental systems and methods used in many studies. Here, indicate whether each material, system or method listed is relevant to your study. If you are not sure if a list item applies to your research, read the appropriate section before selecting a response.

Materials & experimental systems

n/a	Involved in the study
☐	☒ Antibodies
☐	☒ Eukaryotic cell lines
☒	☐ Palaeontology and archaeology
☐	☒ Animals and other organisms
☒	☐ Clinical data
☒	☐ Dual use research of concern

Methods

n/a	Involved in the study
☒	☐ ChIP-seq
☒	☐ Flow cytometry
☒	☐ MRI-based neuroimaging

Antibodies

Antibodies used

SARS-CoV-2 Nucleocapsid antibody (anti-NP antibody)
Antibodies Online (https://www.antibodies-online.com/antibody/6953059/anti-SARS-CoV-2+Nucleocapsid+SARS-CoV-2+N+antibody/)
Cat No: ABIN6953059
Dilution 1/500

Alexa Fluor anti-rabbit 488
Molecular Probes (https://www.thermofisher.com/antibody/product/Goat-anti-Rabbit-IgG-Fc-Cross-Adsorbed-Secondary-Antibody-Polyclonal/A78953)
Cat No: A78953
Dilution 1/2000

Validation

SARS-CoV-2 Nucleocapsid antibody (anti-NP antibody)
Antibodies Online
Validated by manufacturer by ELISA and WB (https://www.antibodies-online.com/antibody/6953059/anti-SARS-CoV-2+Nucleocapsid+SARS-CoV-2+N+antibody/)

Alexa Fluor anti-rabbit 488
Molecular Probes
Validation statement on manufacturers webpage: "This Antibody was verified by Peptide array to ensure that the antibody binds to the antigen stated." (https://www.thermofisher.com/antibody/product/Goat-anti-Rabbit-IgG-Fc-Cross-Adsorbed-Secondary-Antibody-Polyclonal/A78953)

Eukaryotic cell lines

Policy information about cell lines and Sex and Gender in Research

Cell line source(s)

293T CRL-3216 cells from ATCC

Vero ACE2/TMPRSS2 and 293T ACE2/TMPRSS2 from Papa G et al. 2021 (Papa, G. et al. Furin cleavage of SARS-CoV-2 Spike promotes but is not essential for infection and cell-cell fusion. PLoS Pathog 17, e1009246, doi:10.1371/journal.ppat.1009246 (2021))

Expi293 from Elegheert J. et al. (Lentiviral transduction of mammalian cells for fast, scalable and high-level production of soluble and membrane proteins. Nat Protoc 13, 2991-3017, doi:10.1038/s41596-018-0075-9 (2018))

Authentication

293T CRL-3216 cells ATCC authenticated by STR profiling
Vero ACE2/TMPRSS2 and 293T ACE2/TMPRSS2 were not authenticated
Expi293 were not authenticated

Mycoplasma contamination

293T CRL-3216 cells were mycoplasma-free
Vero ACE2/TMPRSS2 and HEK293T ACE2/TMPRSS2 cells were were mycoplasma-free
Expi293 were mycoplasma-free

Commonly misidentified lines
(See ICLAC register)

No commonly misidentified cell lines were used

Animals and other research organisms

Policy information about studies involving animals; ARRIVE guidelines recommended for reporting animal research, and Sex and Gender in Research

Laboratory animals	Male K18 hACE2 mice (strain 2B6.Cg-Tg(K18-ACE2)2PRl,m/J) were purchased from Charles River (18-20g). Mice were housed in groups of 4 to 5. Photoperiod = 12 on:12 off dark/light cycle. Ambient animal room temperature is 21 C, controlled within ± 2 C and room humidity 50% ± 5%.
Wild animals	Not applicable
Reporting on sex	Sex was not considered in study design.
Field-collected samples	Not applicable
Ethics oversight	Murine challenge studies were conducted in accordance with UK Home Office Scientific Procedures Act (ASPA, 1986) and project licence PP4715625 has been approved by the University of Liverpool AWERB.

Note that full information on the approval of the study protocol must also be provided in the manuscript.
